# Deep residual-dense network based on bidirectional recurrent neural network for atrial fibrillation detection

**DOI:** 10.1038/s41598-023-40343-x

**Published:** 2023-09-13

**Authors:** Asif Ali Laghari, Yanqiu Sun, Musaed Alhussein, Khursheed Aurangzeb, Muhammad Shahid Anwar, Mamoon Rashid

**Affiliations:** 1 Software College, Shenyang Normal University, Shenyang, 110034 China; 2 Liaoning University of Traditional Chinese Medicine, Shenyang, China; 3 Department of Computer Engineering, Faculty of Science and Technology, Vishwakarma University, Pune, 411048 India; 4 Department of Computer Engineering, College of Computer and Information Sciences, King Saud University, P.O. Box 51178, Riyadh, 11543 Kingdom of Saudi Arabia; 5 Department of AI and Software, Gachon University, Seongnam-si, 13120 South Korea

**Keywords:** Cardiology, Diseases, Computer science

## Abstract

Atrial fibrillation easily leads to stroke, cerebral infarction and other complications, which will seriously harm the life and health of patients. Traditional deep learning methods have weak anti-interference and generalization ability. Therefore, we propose a new-fashioned deep residual-dense network via bidirectional recurrent neural network (RNN) model for atrial fibrillation detection. The combination of one-dimensional dense residual network and bidirectional RNN for atrial fibrillation detection simplifies the tedious feature extraction steps, and constructs the end-to-end neural network to achieve atrial fibrillation detection through data feature learning. Meanwhile, the attention mechanism is utilized to fuse the different features and extract the high-value information. The accuracy of the experimental results is 97.72%, the sensitivity and specificity are 93.09% and 98.71%, respectively compared with other methods.

## Introduction

According to the WHO 2020 World Health Statistics report, an estimated 41 million people worldwide died from non-communicable diseases in 2016, accounting for 71% of total deaths. The leading cause of death remains cardiovascular and cerebrovascular diseases, accounting for 44% of NCDS. According to relevant statistics, about 540,000 people died of sudden cardiac death in China every year, and the lack of certain first-aid measures has made this kind of disease a major problem threatening human life and health^1,2^.

A large proportion of patients with atrial fibrillation (AF) do not have obvious symptoms at the beginning of the onset, because atrial fibrillation has a certain degree of occultation. Such concealment is often a potential hazard of aggravation and complications^3^. As a result, atrial fibrillation is usually only detected during routine physical examinations. When considering the 12 lead ECG electrocardiogram (ECG) for the complexity of operation, professional, it is necessary to apply the intelligent detection algorithm of atrial fibrillation to portable mobile medical equipment and real-time monitoring of atrial fibrillation^4^, and more and more scientific research institutions have paid attention to it. The early portable intelligent diagnosis of atrial fibrillation mainly refers to the mobile medical equipment for the atrial fibrillation detection, which combines the portable hand-held acquisition device of ECG signal with the intelligent detection algorithm.

Recently, with the pullulation of technology and the progress of the electronics industry, as well as the general increase of people's health awareness, mobile health has seen rapid development. Through short time acquisition of single lead mobile ECG signal, combined with intelligent analysis algorithm, the occurrence of atrial fibrillation can be timely and effectively captured, and the early detection of atrial fibrillation can be realized^5^. Before the equipment is put into widespread use, the study of accurate atrial fibrillation detection algorithm is particularly important.

At the same time, compared with the traditional AF detection algorithm based on feature extraction, in recent years, more and more studies began to apply deep learning network to AF detection and recognition. The advantage of deep learning network mainly lies in that it can omit the complicated feature extraction steps and directly simplify the original multi-step detection into the end-to-end network AF detection. And it can provide good generalization ability for the application of atrial fibrillation detection in the field of portable mobile medicine. Therefore, for the portable medical real-time ECG monitoring scenario, the early detection algorithm of atrial fibrillation based on deep learning models is the focus in this article.

Our main contributions are as follows. Residual dense CNN is applied to the early detection of atrial fibrillation. Considering that ECG signal is one-dimensional time series signal, residual dense CNN and RNN are combined in this study, which improves the accuracy of early detection of atrial fibrillation while optimizing the network structure, making it more applicable to the early detection scene of atrial fibrillation based on timing signals. The model is divided into two main parts: residual dense CNN, which is used to extract local features from the original ECG signal and can further compress long sequence data; RNN, which is used to extract global features from the original ECG signal, is good at describing time series. Feature fusion of Residual dense CNN can optimize the automatic classification algorithm of ECG data.

## Related works

With the continuous deepening of computer technology revolution and the continuous development of artificial intelligence, computer-aided medical diagnosis technology has gradually matured in the exploration of many scholars. In the early years, the pattern recognition method based on ML has been extensively applied in the study of ECG signal classification^6,7^, which mainly includes four major steps: pretreatment, waveform detection, feature extraction and arrhythmia classification. Firstly, the influence of various noises and pseudo-signals is eliminated through signal processing methods such as filtering, followed by feature extraction. Finally, classification is completed through various algorithms. Feature extraction includes mean value, principal component analysis, Fourier transform, wavelet transform, etc. The classifiers mainly include SVM, ADT, neural network, GBDT, sample drop coefficient (CosEn) and LDA. For example, Iscan et al.^8^ used Gaussian mixture model with P-wave characteristics to classify ECG signals, achieving 98.10% sensitivity and 91.70% specificity. Labate et al.^9^ used wavelet decomposition to decompose ECG signals into signals in different frequency domain segments, and then extracted the peak average power ratio feature values of signals in each frequency domain segment and input them into SVM classifier for classification. Polat et al.^10^ used a support vector machine-based way to detect four arrhythmias, including atrial fibrillation, using the 2017 PhysioNet Challenge training set. Its model training achieved 86.23% accuracy and validation accuracy of 87.71%. Rahimi et al.^11^ proposed an algorithm for single-lead ECG atrial fibrillation detection based on heart rate variability and spectral characteristics. Papaioannou et al.^12^ proposed a new short-time series drop estimation method, which achieved 91% mean sensitivity and 98% specificity in the classification. In the feature extraction method proposed by Diker et al.^13^, wavelet transform was firstly used to de-noise signals, and then the positions of R wave, Q wave, S wave, T wave and P wave were extracted respectively to further reduce computational complexity. The optimal accuracy of this algorithm was 96.79%. Xiong et al.^14^ explored Entropy-AF droplet measurement method and tested data collected by wearable ECG, obtaining accuracy of 87.10%, sensitivity of 92.77%, and specificity of 85.17%. Traditional methods have the advantage of explicability, but they are relatively weak in self-learning, usually unable to learn potential abstract patterns, requiring sufficient manual intervention and spending a lot of time on feature extraction and feature selection. Meanwhile, it has weak generalization ability when facing noise and individual difference problems in reality.

In addition, deep learning methods such as CNN have achieved initial success in ECG data processing, which provides another opportunity to further improve the scalability and accuracy of automatic classification of ECG signals. CNN model has two significant advantages. First, raw ECG signal is used as the input data of end-to-end deep CNNs (DNNs), which does not need to be preprocessed by manual rules. Second, according to appropriate learning and training set data, classification types can be continuously expanded. According to different network structures, the original data are abstracted layer by layer and transformed into the final feature representation required by classification tasks, which overcomes the limitations of the traditional machine learning algorithms with independent input and output.

In recent years, some new attempts have been made in DNNs, such as residual block, deep convolutional neural network, deep residual neural network, RNN and deep LSTM. In order to effectively select feature information and enhance the interpretability of models, the attention mechanism has been paid attention to in the classification of arrhythmias. Hua et al.^15^ designed an 11-layer one-dimensional CNN model for the classification and detection of normal and myocardial infarction (MI) signals in single-lead ECG, with an average accuracy of 93.53% and 95.22%, respectively. Chen et al.^16^ converted continuous ECG signals into RR interval data and transmitted it to an end-to-end model combining multi-layer CNN and RNN, achieving an accuracy of 87.4% on the validation set. Alhussainy et al.^17^ compared two CNNs with three layers and two layers, using short-term Fourier transform and stationary wavelet transform, and obtained 98.29% accuracy. Wu et al.^18^ proposed a magnetic resonance imaging-oriented novel attention-based glioma grading network. By applying the dual-domain attention mechanism, both channel and spatial information could be considered to assign weights, which benefited highlighting the key modalities and locations in the feature maps. Li et al.^19^ proposed a computer aided diagnosis (CAD) model Cov-Net for accurate recognition of COVID-19 from chest X-ray images via machine vision techniques^20^. Liao et al.^21^ proposed a COVID-19 prediction model based on time-dependent SIRVD by using deep learning. This model combined deep learning technology with the mathematical model of infectious diseases, and forecasted the parameters in the mathematical model of infectious diseases by fusing deep learning models such as LSTM and other time prediction methods. Liu et al.^22^ developed a new dataset of cervical cytology images named Cx22, which consisted of the completely annotated labels of the cellular instances based on the open-source images. Din et al.^23^ presented the critical analysis of the research and findings already done to detect and classify BC using various imaging modalities including “Mammography”, “Histopathology”, “Ultrasound”, “PET/CT”, “MRI”, and “Thermography”. Liu et al.^24^ showed an intelligent visual servo control algorithm based on Q-learning to generate distance-directed end effector locomotion.

There are also more researches on single-lead atrial fibrillation detection approaches based on deep learning. Luo et al.^25^ realized multi-classification detection of cardiac arrhythmia including atrial fibrillation by using convolutional recurrent neural network and ECG signal data of single lead, and achieved good detection effect. Sabut et al.^26^ collected a large amount of ECG data by using a single-lead mobile ECG device and constructed a 34-layer convolutional neural network. Finally, a multi-classification detection of 12 arrhythmia diseases including atrial fibrillation was realized by Softmax classifier. 2019, Wu et al.^27^ set up a network structure with DNN, applied it to build a large single guide for self-use LianXin database, realized arrhythmia classification including atrial fibrillation.

## Proposed atrial fibrillation detection method

**Figure 1:**
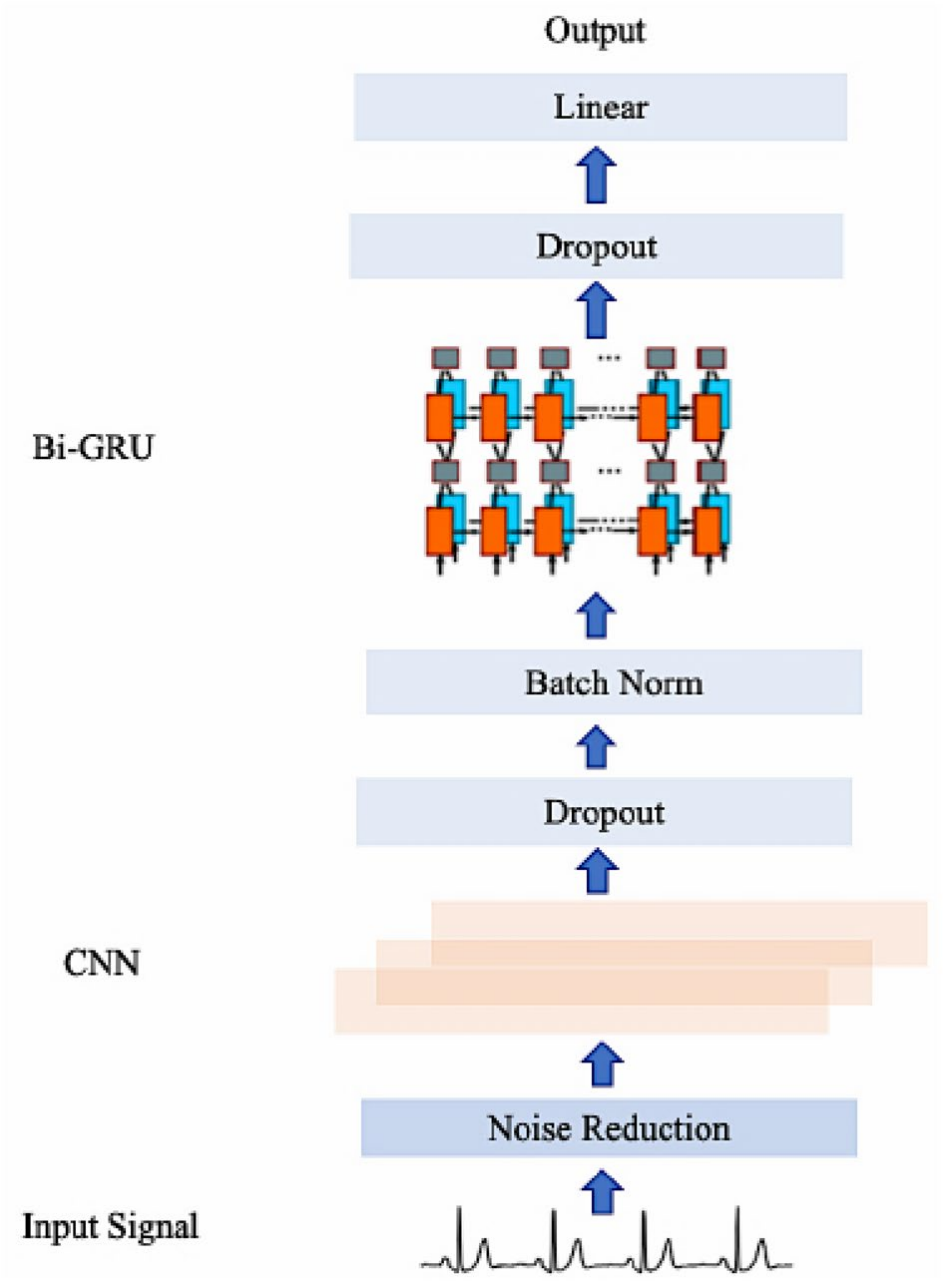
Proposed network.

Compared with some traditional ML methods, the significant advantage of deep learning is that it can omit the steps of manual extraction or feature selection, and automatically learn features through models. Its goal is to find features with good representational ability. In the process of network training, features will be processed by repeated comprehensive calculation. The deeper network has the better ability to train features for representation. The depth of the output feature graph itself will increase with the increase of network layers, thus increasing the number of available features. Therefore, the depth of neural network is crucial in the study of deep learning. However, as the depth increases, problems such as gradient explosion, gradient dissipation, and network performance "degradation" will occur. Deep residual network and dense convolution neural network are generated to ensure network depth and avoid corresponding defects or risks caused by increasing network depth while continuously making full use of features. Our proposed method is as displayed in Fig. 1.

**Figure 2:**
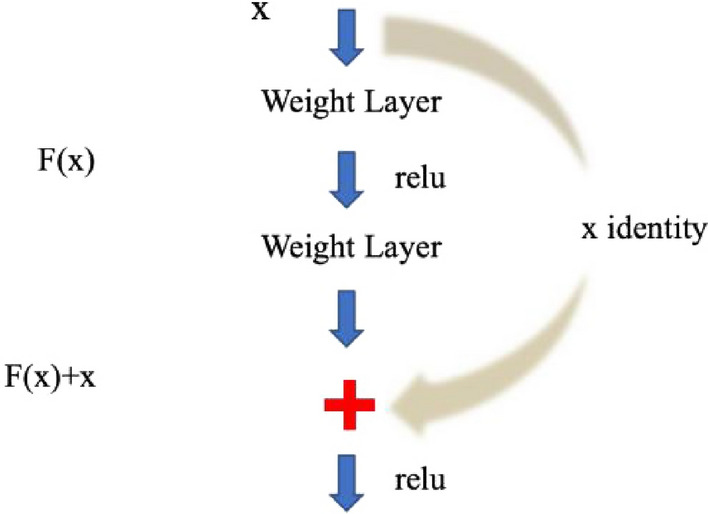
Residual flow chart.

ResNet solves the gradient problem and "degradation problem" by introducing a new residual structure, allowing the continuous deepening of network depth. The residual structure is to establish a shortcut connection between the inputs and outputs of each layer, as shown in Fig. 2. This connection enables subsequent new layers to learn new features based on the input from the previous layer, that is, to learn residuals.

The residual structure in Fig. 2 provides two mapping modes between different weight layers, thus achieving the identity mapping when the network is optimized. This identity mapping is achieved by providing two options, one is identity mapping and the other is residual mapping, the residual between the input and output is F(x). When the network is optimal, the residual block is 0 as the network deepens, which keeps the network in the optimal state. When the internal features of the network are optimal, the subsequent layers remain unchanged, that is, the solution space of shallow network is a subset of the solution space in the deep network, thus solving the "degradation" problem.

**Figure 3:**
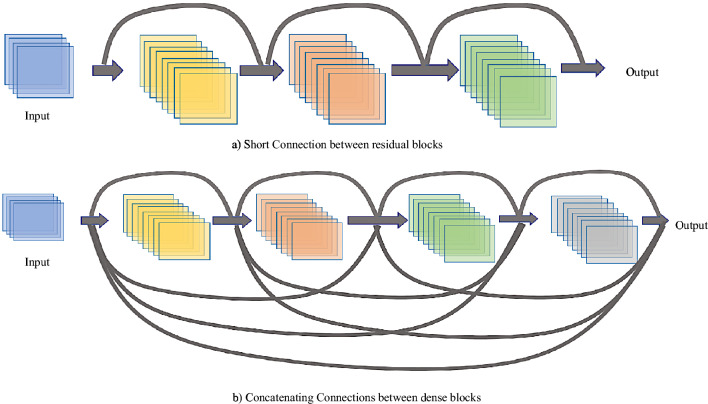
Comparison between ResNet and DenseNet connection.

Dense connection neural network is a new CNN model based on ResNet network. ResNet solves the problem of network depth. While Inception network proposed by Google is a deep learning network focusing on network width. The feature of DenseNet is to enhance feature in the training process by changing the connection mode between network layers, so that features can be utilized to the utmost while reducing the number of network parameters. Its main realization principle is to achieve maximum information transmission between layers by directly connecting all layers of the network, thus strengthening the transmission of features and realizing the maximum utilization of features. Both ResNet fast connection mode and DenseNet dense connection mode solve the problem of gradient disappearance, while DenseNet improves network efficiency and has fewer parameters through feature reuse. The comparison of the two connection modes is shown in Fig. 3.

The premise of the success of deep learning-based atrial fibrillation detection algorithm is that there are enough labeled data for training. In this study, 6877 ECG signal data are divided into training set, validation set and testing set in the ratio of 6:2:2, containing 4125, 1375 and 1377 data pieces respectively. In the training process of the deep learning model, the method of gradient descent is used for continuous iteration to obtain the optimal solution, so that the final output results can be fitted with the training data to the maximum extent, and the loss function is gradually reduced until the network converges. In this paper, the SGD method is used for Gradient updating. Compared with the BGD (Batch Gradient Descent) method, SGD will perform gradient update on samples one by one at each update. It is more suitable for data sets with large data volume, and it has no redundancy and faster update speed.

**Figure 4:**
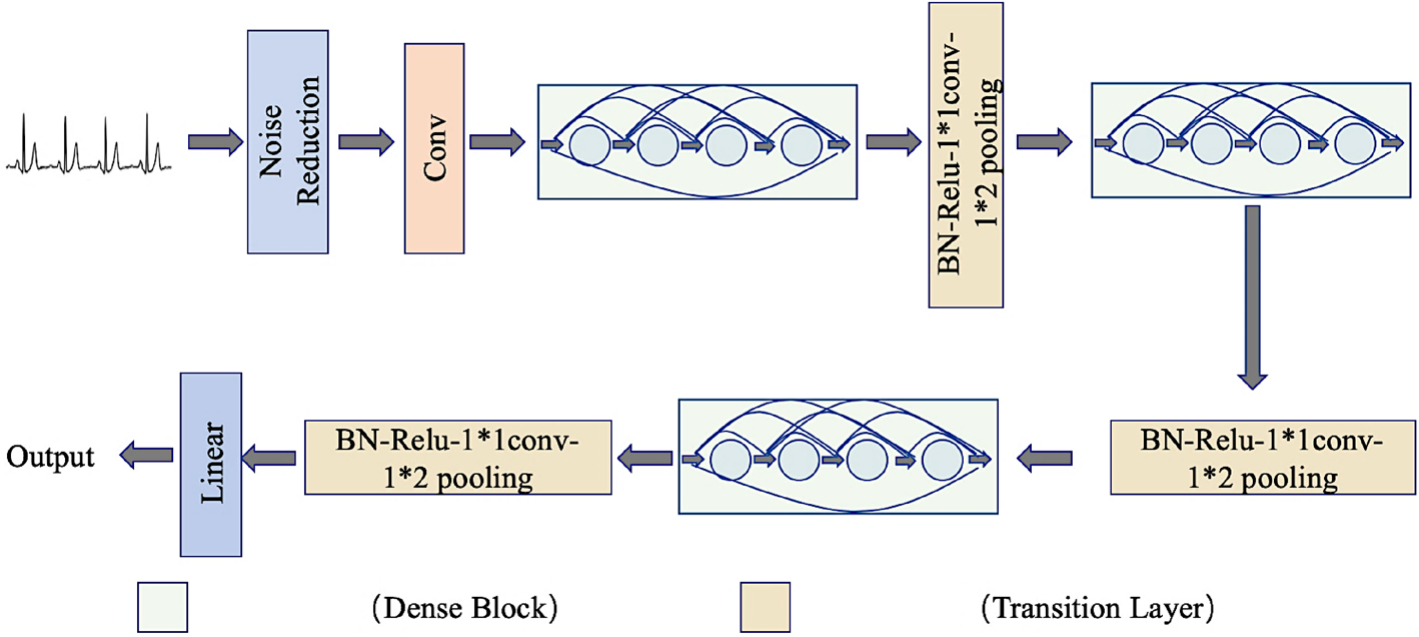
Schematic diagram of the one-dimensional DenseNet.

The one-dimensional DenseNet network constructed in this paper is composed of several Dense blocks and Transition Layer. The denoising processing module is added after the initial ECG signal input. After a layer of one-dimensional convolution, the combination of three intensive modules and transition modules is continuously passed. After the model training is completed, AF discrimination results are output through linear classifier, as shown in Fig. 4.

**Figure 5:**
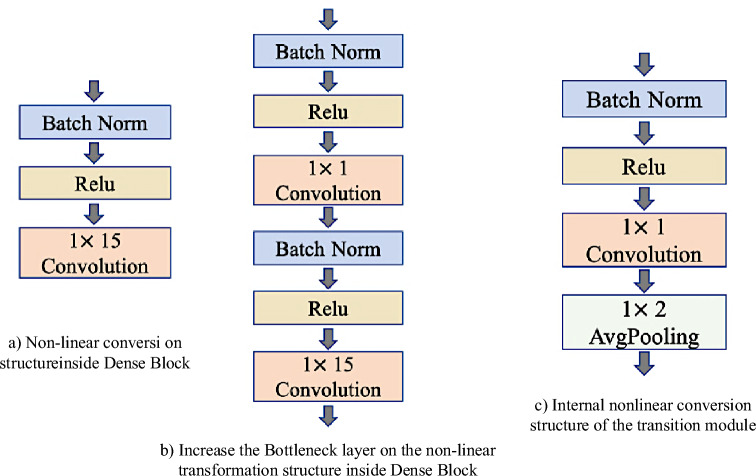
Internal structure of each module in the one-dimensional DenseNet.

As can be seen from Fig. 5, each dense module contains many Dense layers, which are densely connected with each other, and the size of feature graphs in each layer is consistent. In addition, in order to ensure the constant size of the feature graph, all step sizes in the convolution are set to 1. Dense modules are connected through transition modules, and their internal structures are shown in Fig. 5c.

In each dense module, the nonlinear combination function is generally composed of Batch normalization (BN), the activation function ReLu and the one-dimensional convolution, as shown in Fig. 5a. In the dense module, k feature graphs will be output after each layer is convolved, that is, the number of channels in the feature graph will increase by k after each layer is convolved. Although the k is generally set to a small value, as the number of layers increasing, the large amount of feature reuse results in large amounts of inputs for subsequent layers. Therefore, a transition layer is introduced between two dense modules, and a Bottleneck layer is introduced inside the dense module to reduce computation. The structure of the transition module is composed of BN, activation function, ReLU, 1 × 1 convolution and 1 × 2 average pooling.

If the initial number of channels is $k_{0}$, then the number of input channels at first layer is $k_{0} + k(l - 1)$. As the layer number increasing, the calculation pressure is too large. Therefore, 1 × 1 convolution is added in the transition module between the dense modules to compress the feature scale. In this paper, the number of channels is reduced to half of the original, and the calculation is further reduced through the average pooling layer of 1 × 2, the size of the feature graph is reduced, and the number of channels input to the next layer is reduced, which can effectively control the model size from being too large. The bottleneck layer is realized by adding a linear combination of 1 × 1 convolution to the original structure as shown in Fig. 4b. The purpose of adding this layer is not only to reduce the dimensionality and reduce the amount of computation by reducing the number of features, but also to integrate the features of each channel.

The disadvantage of the traditional LSTM model is that it cannot accurately capture the future information and only processes the forward data. Different from LSTM, Bi-LSTM is a recurrent neural network with input layer, two hidden layers and one output layer. The outputs of the positive and negative LSTM layers are combined into a locally focused global eigenvector. Bi-LSTM is able to take full account of the global information hidden in the input data because the module structure can evaluate both past and future input information in a time step. In this paper, the number of LSTM elements in Bi-LSTM module of the model is 128, which means that the length of the global feature vector of each local focus is 128.

The Attention Mechanism is a structural optimization module that simulates the attention of the human brain. Combined with RNN model, it is applied to image classification successfully. Its essence is to use the probability distribution of attention to control the weight parameters of elements in the input data and generate differentiated feature output, so as to optimize the model and make it make more accurate judgment. The attention mechanism can be described as a mapping from a Query to a series of key-value pairs. The calculation of attention in this mechanism has three steps. The first step is to obtain the relevant weight by calculating the similarity between Query and Key. Commonly used similarity calculation formulas include multiplication (1), cascade (2) and perceptron (3). The second step is normalization through the Softmax function. Finally, the final attention vector output is obtained by summing weights and corresponding keys.

1 $$f(Q,K_{i} ) = Q^{T} W_{\alpha } K_{i}$$

2 $$f(Q,K_{i} ) = W_{\alpha } [Q:K_{i} ]$$

3 $$f(Q,K_{i} ) = v_{\alpha }^{T} \tanh (W_{\alpha } Q + U_{\alpha } K_{i} )$$

where $W_{\alpha }$, $U_{\alpha }$, $v_{\alpha }$ are learn-able parameters. Q refers to queries, and K refers to key value.

## Experiments and analysis

### Evaluation index

The AF detection algorithm in this paper is a binary task. 0 represents non-AF sample and 1 represents AF samples. Therefore, in this paper, the binary evaluation method will be used to evaluate the results, which will be used as the standard for selecting the hyperparameters of the network model and comparing with other research results. At the same time, the final loss of network test sets is compared when the results are compared.

In this paper, the cross entropy function is used as the loss function to calculate the loss of the training process and the final test set. The loss function calculation formula of a single sample is shown in Eq. (4).

4 $$L = - [y\log \hat{y} + (1 - y)\log (1 - \hat{y})]$$

Cross entropy loss function is a common loss function in binary classification model. From the perspective of maximum likelihood, the conditional probability of sample label 0 and sample label 1 is integrated, as shown in formula (5). In order to maximize the probability value of $P(y|x)$ and ensure the monotonicity of the function, *log* function is introduced to make $Loss = - \log P(y|x)$. The Loss function of a single sample in formula (4) can be obtained. In this paper, for a large number of sample problems based on deep learning, the complete loss function can be obtained by stacking the losses of N samples, and the final loss of each training or test set can be calculated.

5 $$P(y|x) = \hat{y}^{y} \cdot (1 - \hat{y})^{1 - y}$$

In addition to the selected cross entropy loss function, this study also selects some quantitative indexes to evaluate the parameter selection and performance of the algorithm. The following major evaluation indexes are mainly used.

#### Accuracy

Accuracy is the most intuitive and best understood index, and its value is the ratio of the number of data correctly classified by the algorithm to the total number of data input to the algorithm. However, this index cannot be used as the only objective evaluation index in the case of extremely obvious data imbalance in data categories or extremely biased data. Therefore, other indexes are needed to comprehensively evaluate algorithm performance.

#### Confusion matrix, sensitivity, specificity

In this study, atrial fibrillation samples are regarded as positive and non-atrial fibrillation samples are as negative. If the instance is positive and is predicted to be positive, it is called true positive (TP). Conversely, if an instance is positive but incorrectly is predicted as negative, it is called pseudo-negative (FN). Similarly, if an instance is negative and is predicted to be negative, it is called true negative (TN). An instance that is negative but predicted to be positive is called a false positive (FP).

6 $$ACC = ((TN + TP))/((TN + TP + FN + FP))$$

7 $$Sensitivity = TP/(TP + FN)$$

8 $$Specificity = TN/(TN + FP)$$

Confusion matrix is a visual tool to show the classification accuracy results. Each column represents the number of predicted categories, and each row represents the number of actual categories. In this study, in addition to the quantitative values of the above indexes, the final confusion matrix of the test set in the data set is used as an intuitive evaluation index of the network results.

### Data set and pre-processing

The data set selected in this study is a 12-lead ECG signal data set published in the China Physiological Signal Analysis Challenge (CPSC2018). For research needs, this study uses the information of single leads in the 12 leads for analysis.

Currently, there are several different publicly available databases in the field for the detection of arrhythmia-like diseases. Among them, MIT-BIH database is the most commonly used database for detecting cardiac arrhythmias. The data in this database is double-lead ECG data, which contains 48 ECG records from 47 patients with a sampling frequency of 360Hz, each of which lasts about 30 min and contains 15 types of arrhythmias. In addition, there is MIT-BIH AF database, which is dedicated to AF detection algorithm research and contains 25 ECG ECG signal records, each of which has a long duration, and most of them are paroxysmal AF.

The ECG signal itself is a weak signal, and at the same time, the data collected by the ECG signal acquisition equipment will contain noise, generally including power frequency noise, human myoelectric interference and baseline drift noise types. Therefore, before the ECG signal is input into the designed deep neural network, it is necessary to pre-process the ECG signal with noise reduction. The common noise reduction methods mainly include filter noise reduction and wavelet noise reduction.

Filter noise reduction is a common noise reduction processing method, which is realized based on frequency domain analysis. The useful signal and noisy signal are separated in the frequency domain mainly through the selected or designed filter, and the required frequency components are obtained from the signal or the unwanted frequency components are removed. Common noise reduction filters generally include low-pass and high-pass filters and bandpass filters. Among them, the low-pass filter and the high-pass filter process the signal in the opposite way, low-pass means that the signal below a certain cutoff frequency passes through the filter, and the signal above the cutoff frequency is weakened or reduced, and the high-pass filter, on the contrary, represses the component below the cutoff frequency, and passes the component higher than the cutoff frequency. The bandpass filter attenuates the frequency components of other components to a very low level through the signal components within a certain frequency segment.

### Network implementation details

Considering that the network scale should be controlled within a small range, and the network advantage of DenseNet is the small model size, the growth rate k refers to the number of k feature channels that will be increased after each convolution layer in DenseNet. Therefore, the growth rate of the network is set to 12 in this study (k = 12). A smaller growth rate is used to effectively control the network size while ensuring network learning ability. At the same time, we set the initial learning rate as 0.1, epoch number as 64, and the number of generation selection as 300. The test results are shown in Table 1.

**Table 1:** Comparison the results of the number of different dense blocks and dense layers in their network.

Number of dense modules	Number of network layers in each module	ACC	Sensitivity	Specificity
3	8	0.9821	0.9314	0.9813
3	16	0.9775	0.9213	0.9797
4	8	0.9714	0.9189	0.9811
4	16	0.9793	0.9142	0.9776
5	8	0.9826	0.9289	0.9824
5	16	0.9783	0.9124	0.9714

From Table 1, it can be seen that with the increase of the layer number, the results will change or have little influence, and the index values will hardly be significantly improved. When the number of intensive modules is 3, the network training is sufficient. Similarly, increasing the number of network layers within each dense module will not improve the results and will make the network more complex. Therefore, in consideration of experimental results and network scale, three dense modules will be used in subsequent experiments, each of which has an 8-layer network structure.

**Figure 6:**
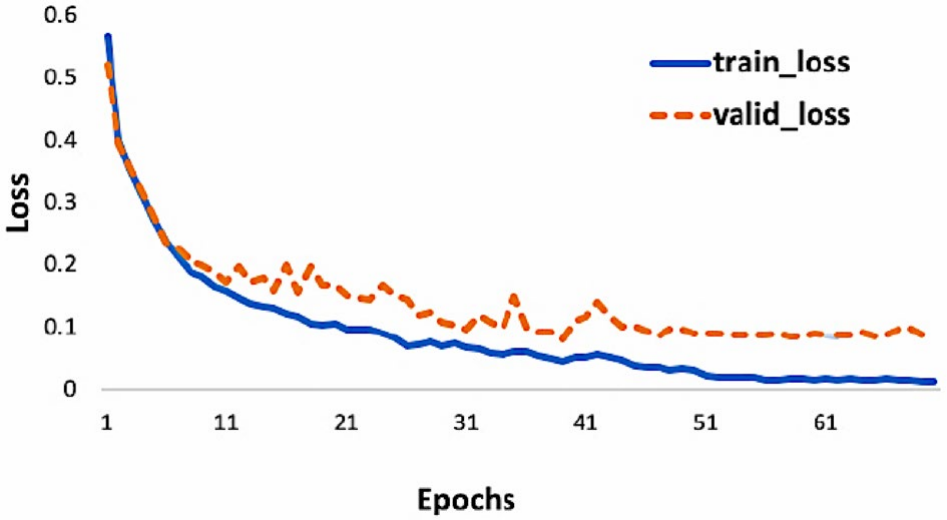
Convergence of the train set and validation set of the 1D DenseNet.

Learning rate determines the speed of adjusting network parameters in the process of gradient descent, which is a very important hyperparameter. In this study, the auto-adjusted learning rate is used, and the initial value of the initial learning rate is set to 0.1. When the training reaches half and three-quarters of the set number of iterations, the current learning rate is multiplied by 0.1 to reduce the learning rate to achieve better optimization effect. However, we can see the training process of the network through the convergence curves of the training set and verification set in Fig. 6. The convergence speed of the network is fast. It basically converges before the 50th substitution. Therefore, the training convergence speed of the network itself is fast, and considering the small scale of the network, it also further proves the superiority of the network itself. Meanwhile, the loss value of the final test set also reached 0.0385, which also reflects the superiority of the network and algorithm in this study.

Considering the rapid convergence of the network, the convergence of the network will be achieved around the 50th generation selection. Therefore, in the following network training, the number of selected generations is set to 100 and the number of processing per batch is 64. The number of intensive modules is 3, and the number of internal network layers of modules is 8. Meanwhile, the initial learning rate is 0.1, and the growth rate k is set to 12.

### Results analysis

**Figure 7:**
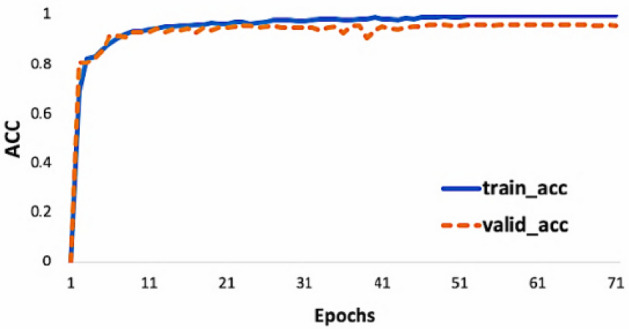
Curves of the accuracy of the 1D DenseNet training and validation sets.

The training convergence process of the network is shown in Fig. 7. It can be seen that the network convergence speed is very fast, and the training set and verification set can basically start to converge around the 50th generation selection. Here, the loss of training set is close to 0, and the loss of verification set begins to stabilize below 0.1 at about 50 times of generation selection. With the training of the network, the accuracy rate increases rapidly in the training process and maintains at a state approaching 1, which further proves the stability and accuracy of the algorithm in this paper.

In this paper, the data of each of the 12 leads ECG are used as network input to verify the algorithm, and the results are shown in Table 2. As can be seen from the results, although the performance results are different, the accuracy of each lead can exceed 95%, which proves that the new algorithm is applicable to the data of each of the twelve leads. Better results are obtained regardless of which lead is used, and atrial fibrillation was best detected on the unipolar limb lead. As can be seen from Table 2, the results on the lead are also very good, which also proves the feasibility of the application of the algorithm in portable mobile medical scenarios.

**Table 2:** Results of one-dimensional DenseNet on each lead of ECG 12 leads.

Lead	Accuracy	Sensitivity	Specificity
1(I)	0.9757	0.8974	0.9885
2(II)	0.9745	0.8945	0.9834
3(III)	0.9763	0.9224	0.9846
4(aVR)	**0.9821**	**0.9355**	**0.9911**
5(aVR)	0.9558	0.8497	0.9689
6(aVF)	0.9794	0.9392	0.9872
7(V1)	0.9693	0.8943	0.9814
8(V2)	0.9682	0.9324	0.9724
9(V3)	0.9764	0.8897	0.9525
10(V4)	0.9592	0.8858	0.9698
11(V5)	0.9514	0.8499	0.9547
12(V6)	0.9663	0.8699	0.9438

Significant values are in bold.

Sensitivity is slightly lower in both networks. It can be seen from the confusion matrix in Table 2 that the sensitivity is slightly lower than the other two indicators because the number of AF samples in the data set is smaller than that of non-AF samples. The two roughly present a sample ratio of 1:5, and the sample proportion is small, so compared with the calculation of sensitivity, the denominator value is relatively small, resulting in slightly less sensitive experimental results.

**Figure 8:**
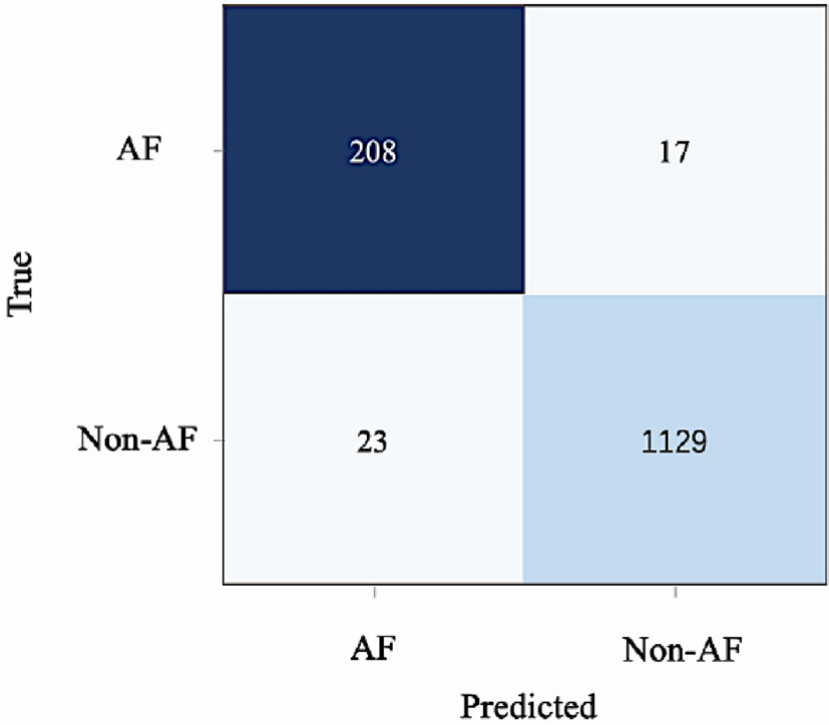
Confusion matrix.

In conclusion, one-dimensional DenseNet achieves high accuracy in the detection of atrial fibrillation. The sensitivity and specificity of the algorithm reach 93.55% and 99.11%, respectively. The very high specificity indicates that the proportion of correctly classified samples in the original negative samples is very high, the classification effect of the network is very good. The confusion matrix also shows the best classification detection result as shown in Fig. 8.

At the same time, we also compare with other advanced methods including CNN-LSTM^28^, OTE^29^. The dataset is CinC2017 Challenge data set. By calculating the total number of True positive (TP), False negative (FN) and False positive (FP), the F1 score of each category is calculated as an indicator to evaluate the performance of the new method. The F score for category C is calculated as follows:

9 $${F}_{1c}=\frac{2TP}{2TP+FP+FN}$$

The average of F1 scores for the normal rhythm class (F_1N_), atrial fibrillation rhythm class (F_1A_), and other rhythm class (F_1O_) is used as the total F1 score, which is calculated as:

10 $$F1=\frac{{F}_{1N}+{F}_{1A}+{F}_{1O}}{3}$$

It is worth noting that although the F1 score for the noise class is not included in the calculation, misclassifying the noise sample into other categories can also affect the final score. Table 3 shows the results of tenfold cross-validation of each model.

**Table 3:** A tenfold cross-validation results of each model.

Model	CNN-LSTM	OTE	Proposed
F1N	0.767	0.756	0.867
F1A	0.198	0.187	0.577
F1O	0.417	0.388	0.584
F1	0.461	0.444	0.642
Accuracy (%)	61.912	62.022	74.503

Maximum F1 is in bold.

**Figure 9:**
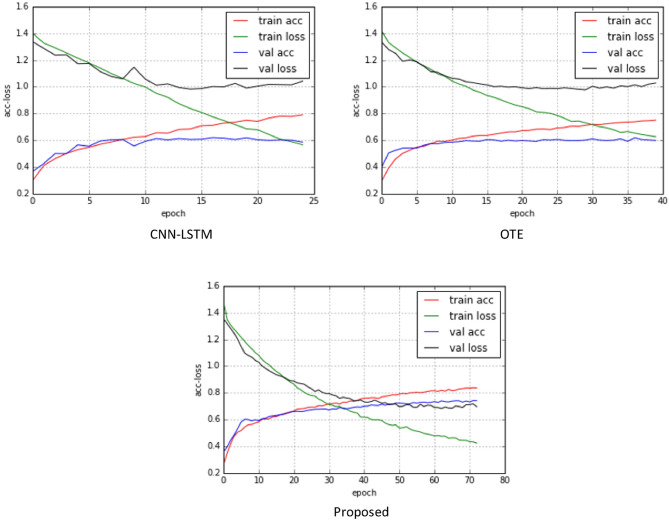
Loss and correctness change of three model training stages. Red line: The model's accuracy in the training set; Green line: Loss of model in training set; Blue line: the accuracy of the model in the verification set; Black line: Loss of the model in the verification set.

From an outcome point of view, the model performs better for categories with more training samples (normal sinus rhythm, other rhythms). From the results, the recognition performance of the model with more samples (normal, other) was better than that of the model with fewer training samples (AF rhythm). As shown in Fig. 9, the loss and correctness changes in the training phase. It is observed that the training loss continues to decline, while the loss of the validation set levels off after about 10 training cycles, and the performance of the model is difficult to improve.

## Conclusion

This paper introduces the basic structure of DenseNet, and compares it with the original representative ResNet network, and analyzes the differences between them. At the same time, the advantages of DenseNet are explained structurally. Its characteristics with small parameter scale, small model size and fast analysis speed make it more suitable for ECG signal analysis of single lead portable mobile devices, and also make the application of the algorithm more possible. The results of the network proposed in this paper are better than one-dimensional DenseNet structure in each index, and its convergence curve reflects its early loss value and convergence process is better than one-dimensional DenseNet network. However, the problem of small oscillations occurred in the early stage of the verification set convergence process, which was caused by the addition of a new bidirectional RNN network structure to the new network structure. The problem of small oscillations with inappropriate learning rate appeared in the initial training process. However, by setting the adaptive learning rate and the good learning ability of the network itself, The loss value converges quickly and becomes stable. In future work, we will use more advanced deep learning methods to obtain better detection results and apply them to clinical medical experiments.

## Data Availability

If the reader needs some data, please contact the corresponding author.
